# Comorbidity Burden and Survival After Primary Surgery for Oral Cavity Squamous Cell Carcinoma: Tumor Stage as an Effect Modifier

**DOI:** 10.3390/cancers18172825

**Published:** 2026-09-01

**Authors:** Mateusz Szewczyk, Krzysztof Przybylski, Bartosz Nowak, Bartosz Wojtera, Dominika Wojnar, Damian Rusek, Jakub Pazdrowski

**Affiliations:** 1 Department of Head and Neck Surgery, Poznan University of Medical Sciences, 61-701 Poznań, Poland; krzysztof.przybylski@wco.pl (K.P.); bartosz.wojtera@wco.pl (B.W.); jakub.pazdrowski@wco.pl (J.P.); 2 Department of Head and Neck Surgery, Greater Poland Cancer Center, 61-866 Poznań, Poland; bartosz.nowak@wco.pl (B.N.); dominika.wojnar@wco.pl (D.W.); 3 Department of Cancer Pathology and Prevention, Greater Poland Cancer Center, 61-866 Poznań, Poland; damian.rusek@wco.pl

**Keywords:** oral cavity cancer, oral squamous cell carcinoma, Charlson Comorbidity Index, comorbidity, overall survival, disease-free survival, tumor stage, effect modification

## Abstract

**Background/Objectives:** To evaluate the association between comorbidity burden, quantified using the Charlson Comorbidity Index (CCI), and overall survival (OS) and disease-free survival (DFS) in patients treated surgically for oral cavity squamous cell carcinoma (OCSCC), and to determine whether this association is modified by tumor stage. **Methods:** We retrospectively analyzed 325 patients treated with primary surgery for OCSCC at a single institution (2009–2021). CCI was scored excluding the index malignancy component and evaluated as a continuous variable. Univariate and multivariable Cox proportional hazards models assessed the association between CCI and OS/DFS, including a formal CCI × stage interaction term. Kaplan–Meier curves stratified by CCI (>4 vs. ≤4) were generated separately in early- and advanced-stage subgroups and compared by log-rank test. **Results:** CCI was significantly associated with OS (HR 1.14 per point, 95% CI 1.04–1.25, *p* = 0.005) but not DFS (HR 1.02, 95% CI 0.92–1.12, *p* = 0.768). A significant CCI × stage interaction was observed (HR 0.82, 95% CI 0.68–0.99, *p* = 0.036): within early-stage patients, an elevated CCI (>4) was associated with a significantly worse 5-year OS (52.9% vs. 72.3%, *p* = 0.009), whereas no such difference was observed in advanced-stage patients (40.9% vs. 42.6%, *p* = 0.56). **Conclusions:** The comorbidity burden was associated with overall but not disease-free survival after surgery for OCSCC. The association differed by tumor stage and was most apparent in early-stage disease. A recurrence-based classification of deaths showed that non-cancer death accounted for a substantially larger share of mortality among early-stage than advanced-stage patients, supporting a competing-mortality mechanism; independently adjudicated cause-of-death data were not available to confirm this directly. CCI may provide additional prognostic information in early-stage OCSCC.

## Simple Summary

Patients with oral cavity cancer often have other health problems in addition to their tumor, and it is not always clear how much these other conditions affect survival. We studied 325 patients treated with surgery for oral cavity cancer and measured their overall burden of other illnesses using a standard scoring tool, the Charlson Comorbidity Index. We found that a higher burden of other illnesses was linked to shorter overall survival but not to a higher chance of the cancer coming back. This link between other illnesses and survival was only seen in patients whose cancer was caught at an early stage; it was not seen in patients with more advanced tumors. These findings suggest that, in early-stage oral cavity cancer, assessing a patient’s overall health may help doctors and patients better understand prognosis, although it should not be used on its own to decide whether to reduce treatment intensity.

## 1. Introduction

Oral cavity squamous cell carcinoma (OCSCC) remains one of the most common malignancies of the head and neck. The most recent subsite-specific analysis estimated 758,000 new cases of lip, oral cavity, and pharyngeal cancer worldwide in 2022, of which oral cavity tumors accounted for approximately 42% [1]; the most recent overall GLOBOCAN update, covering 2024, estimated there were 20.6 million new cancer cases worldwide across all sites, underscoring the continued scale of the global cancer burden within which oral cavity cancer remains a major head and neck malignancy [2]; and nationally, Poland is among the countries with the highest cancer mortality in Europe, underscoring the relevance of institutional data such as ours to the local burden of disease [3]. Despite advances in surgical techniques and adjuvant therapies, prognosis remains closely tied to tumor stage at diagnosis, and established oncologic risk factors—stage, nodal status, perineural invasion (PNI), lymphovascular invasion (LVI), and extranodal extension (ENE)—continue to dominate treatment planning and prognostic discussions in this disease [4].

Oncologic factors, however, only capture part of the prognostic picture. OCSCC disproportionately affects older patients with substantial comorbid disease burdens, and the Charlson Comorbidity Index (CCI)—originally developed to predict 1-year mortality in general medical inpatients [5]—has become one of the most widely used tools for quantifying this burden in oncology. Its application to cancer cohorts, however, is not always methodologically deliberate; a systematic review by Borrelli et al. (2023) found that only two of 115 observational cancer studies applied CCI in a manner matching its original validation outcome, and that the cancer type matched the validation population in just 16 of 115 [6], a critique echoed by the index’s original developer, Charlson et al. (2022), in a subsequent clinimetric review [7].

A pattern reported across several tumor types is that CCI often predicts overall survival more consistently than cancer-specific or disease-free survival—a pattern compatible with a competing-mortality mechanism, whereby comorbidity shortens life expectancy independent of, rather than through, oncologic control. This dissociation has been demonstrated at a meta-analytic level in prostate cancer [8] and within head and neck cancer specifically, including a nationwide Danish cohort of over 25,000 HNSCC patients [9] and a meta-analysis of laryngeal squamous cell carcinoma [10]. However, this pattern is not universal: a nationwide cohort study comparing initial surgery and primary radiotherapy in cT1–2N0M0 OCSCC found that a high CCI was independently associated with worse 5-year disease-specific survival, as well as with overall survival, through multivariable analysis [11]; meanwhile, other large nationwide studies restricted to early-stage (cT1N0M0 and cT2N0M0) OCSCC have likewise reported an elevated CCI as a characteristic of patients with poorer prognosis, even where CCI was analyzed as a baseline covariate rather than a formally tested independent multivariable predictor of disease-specific survival [12,13]. Within oral cavity cancer more broadly, CCI has been independently associated with survival in several population-based and institutional cohorts [14,15,16,17], though its relationship to disease-specific or recurrence-free outcomes in this tumor site varies across cohorts and analytic approaches and has been less consistently examined than its relationship to overall survival.

A further complication is that CCI is strongly correlated with patient age, and age-adjusted formulations (ACCI) outperform the unadjusted score in oral and laryngeal cancer cohorts [18,19], raising the question of how best to disentangle comorbidity’s independent contribution from age itself.

Less well-characterized is whether comorbidity’s prognostic weight is uniform across disease stages or is greatest in patients whose oncologic risks are otherwise low. In advanced oral cancer, comorbidity appears to influence outcomes indirectly by delaying diagnosis or constraining treatment options rather than through a direct prognostic effect independent of treatment received [20], thereby predicting attenuation of any direct CCI–survival association once oncologic risk factors dominate. An analogous stage-stratified approach in early-stage non-small-cell lung cancer supports isolating comorbidity’s effect within a stage-restricted population [21]. If comorbidity’s prognostic value is concentrated in early-stage disease, this would have direct implications for treatment-intensity decision-making, with precedents in oral cancer [22] as well as other tumor types [23,24].

To the best of our knowledge, no prior study in oral cavity cancer has formally tested comorbidity burden as an effect-modified prognostic factor across disease stages using an explicit statistical interaction term, as opposed to stratified or subgroup comparisons alone; nor has any prior study addressed the collinearity between age and CCI through explicit interaction modeling rather than simple covariate adjustment. The specific contribution of the present study is therefore this formal CCI × stage interaction test, and the accompanying handling of age–CCI collinearity, within a surgically treated oral cavity cancer cohort.

### Aim

The aim of this study was to determine whether comorbidity burden, quantified using the Charlson Comorbidity Index, is associated with overall and disease-free survival in patients undergoing primary surgery for oral cavity squamous cell carcinoma, and to test whether this association is modified by tumor stage. We hypothesized that CCI would predict overall but not disease-free survival, consistent with a competing-mortality mechanism, and that the prognostic effect of comorbidity would be greatest in early-stage disease.

## 2. Materials and Methods

### 2.1. Study Population

This was a retrospective cohort study conducted at the Department of Head and Neck Surgery, Poznan University of Medical Sciences and Greater Poland Cancer Center, Poznań, Poland, covering patients treated between January 2009 and December 2021. The study was approved by the Local Bioethics Committee of Poznan University of Medical Sciences (protocol code 419/25) and was conducted in accordance with the Declaration of Helsinki.

Patients were eligible for inclusion if they had histologically confirmed first primary oral cavity squamous cell carcinoma (OCSCC)—that is, OCSCC presenting as the index malignancy, with no prior head and neck malignancy—were treated with primary surgery with curative intent at the study institution during the accrual period, and had complete data available for the Charlson Comorbidity Index (CCI), AJCC stage, and survival outcomes (OS and DFS). Patients with distant metastatic disease (M1) at diagnosis were excluded, as were patients with missing data for the CCI, stage, or survival outcomes. Of 326 patients meeting the inclusion criteria, 325 had complete data available and were retained for the present analysis, which examines comorbidity burden (CCI) and its interaction with tumor stage as predictors of overall and disease-free survival in this cohort. Clinical, pathological, and follow-up data were abstracted from institutional medical records by two investigators (B.W. and B.N.).

### 2.2. Preoperative Workup, Surgical, and Adjuvant Treatment Protocols

Preoperative staging workup included clinical examination, panendoscopy where indicated, and cross-sectional imaging of the head and neck (CT and/or MRI), with chest imaging performed to exclude distant disease; the clinical stage was based on clinical examination and CT and/or MRI findings. Disease was staged according to the 7th edition of the AJCC Cancer Staging Manual, applied consistently across the entire 2009–2021 accrual period; tumor and nodal categories are reported in Table 1 and were used throughout the analysis to reflect pathological (postoperative) staging based on the surgical specimen. The depth of invasion (DOI) was not systematically available for patients treated before 2018 and was therefore not incorporated into staging or analysis, consistent with the continued use of the 7th, rather than 8th, edition criteria throughout the cohort. All patients underwent primary surgical resection targeting a minimum of 1 cm macroscopic tumor-free lateral and deep margin. Intraoperative frozen-section margin assessments were performed for doubtful margins, with re-resection undertaken when frozen-section margins were positive. Neck dissection encompassed levels I–III for laterally located tumors with a clinically and radiologically node-negative (cN0) neck on CT and/or MRI, bilateral neck dissection for midline tumors, and levels I–V for the clinically node-positive (cN+) neck. All patients were evaluated postoperatively by the institutional multidisciplinary tumor board (MDT), comprising head and neck surgeons, radiation oncologists, medical oncologists, and pathologists, which made the final decision regarding adjuvant treatment. Adjuvant radiotherapy was recommended for patients with pT3–T4 disease, close surgical margins (1.0–4.9 mm), positive lymph nodes, or histopathologic evidence of perineural or lymphovascular invasion, consistent with standard head and neck oncology guidelines [4]. The standard radiotherapy protocol delivered 60–66 Gy in 2.0 Gy daily fractions, Monday through Friday, over 6–7 weeks. Adjuvant chemoradiotherapy, substituting concurrent single-agent cisplatin (100 mg/m^2^ every 3 weeks) for radiotherapy alone, was indicated for positive surgical margins or extranodal extension. Post-treatment follow-up was conducted every 3 months during the first year, every 4 months during the second year, every 5 months during the third year, and every 6 months thereafter until death or loss to follow-up. The study database was locked for the original analysis in December 2023; the final statistical analysis, incorporating additional sensitivity analyses conducted during peer review, was completed in June 2026.

### 2.3. Variables and Definitions

Comorbidity burden was quantified using the Charlson Comorbidity Index (CCI), collected at the time of diagnosis. CCI was scored excluding the point(s) allocated for the index malignancy itself (the “solid tumor” component, scored at 2 points for localized disease or 6 points if distant metastases are present at diagnosis). This tier depends on M-status and not nodal (N) involvement; since M1 patients were excluded, this cohort is M0 throughout, so the component would have contributed a fixed 2 points across all patients regardless of disease burden. It was nonetheless excluded so the measure reflects comorbid disease independent of the index cancer, as well as to avoid conflation with stage, which is analyzed separately as the focus of the central CCI × stage interaction test. Disease stage was dichotomized as early (I–II) versus advanced (III–IV) according to AJCC staging. Overall survival (OS) was defined as the time from surgery to death from any cause or last follow-up, while disease-free survival (DFS) was defined as the time to recurrence (local, regional, or distant) or second primary malignancy, whichever occurred first, or last follow-up; second primary malignancies were counted as DFS events because their occurrence, like recurrence, ends the disease-free interval, even though they represent a biologically distinct event from recurrence of the index tumor. Median follow-up was estimated using the reverse Kaplan–Meier method to account for informative censoring by death, given that the majority of patients died during the observation period.

### 2.4. Statistical Analysis

CCI was evaluated as a continuous variable. Univariate Cox proportional hazards regression assessed the association between CCI and OS/DFS. Because age and CCI were strongly correlated (Pearson *r* = 0.80), the potential impacts of shared prognostic information between these variables were explicitly considered in model interpretations. The primary interaction model included CCI, age, advanced-stage status, and a CCI × advanced-stage interaction term; age and CCI were not modeled as an unadjusted additive pair to avoid conflating their effects with the interaction of interest. Variance inflation factors for CCI and age in the primary interaction model were 3.96 and 2.80, respectively (tolerance 0.25 and 0.36), below conventional thresholds for problematic collinearity (VIF > 5). Higher VIFs were observed for advanced-stage status and the CCI × advanced-stage term (8.5–9.5) when CCI was entered uncentered; mean-centering CCI before forming the interaction term reduced these to 1.0–2.0, and centered CCI was used in the final interaction models.

To test whether the prognostic effect of comorbidity depends on disease stage, we fitted a multivariable Cox model for OS, including CCI, advanced-stage status, a CCI × advanced-stage interaction term, and age, with a secondary model additionally adjusted for sex, PNI, LVI, and ENE. Kaplan–Meier curves stratified by CCI (median split, CCI > 4 vs. ≤4) were generated separately within early- and advanced-stage subgroups, with between-group comparisons performed by log-rank tests. The same analytical framework was applied to DFS as a complementary oncologic endpoint to assess whether the prognostic association of CCI with overall mortality was mirrored in disease recurrence risk. The proportional hazards assumption was assessed using Schoenfeld residuals; it was satisfied for all covariates in the primary model (all *p* > 0.05) and for all but PNI (*p* = 0.040) in the fully adjusted model.

All statistical analyses were performed using R (version 4.3; R Foundation for Statistical Computing, Vienna, Austria), with the survival package used for Cox regression, Kaplan–Meier estimation, and Schoenfeld residual testing, and the car package used for variance inflation factor calculations. A two-sided *p*-value < 0.05 was considered statistically significant. This study is reported in accordance with the REMARK (REporting recommendations for tumor MARKer prognostic studies) guidelines for prognostic marker studies [26]; a completed REMARK checklist is provided as Supplementary Table S4.

## 3. Results

### 3.1. Patient Characteristics

Of 325 patients, 210 (64.6%) were male, with a mean age of 59.1 ± 12.1 years at diagnosis. Mean CCI was 4.48 ± 1.64 (range 2–9; median 4). Stage distribution was I, 77 (23.7%); II, 79 (24.3%); III, 73 (22.5%); and IV, 96 (29.5%), with 156 (48.0%) early-stage and 169 (52.0%) advanced-stage patients. Mean CCI did not differ meaningfully between early- and advanced-stage groups (4.51 vs. 4.45), indicating that the comorbidity burden was balanced across the stages at baseline. Median follow-up, estimated using the reverse Kaplan–Meier method, was 89 months (95% CI 80–97). During follow-up, 191 patients (58.8%) died and 147 (45.2%) experienced a DFS event (132 true recurrences and 15 second primary malignancies, per the DFS definition in Section 2.3). Because observed follow-up time is truncated by death for patients who died, we additionally report crude observed time separately by vital status: among the 134 patients alive at last follow-up, the observed follow-up was 79.8 ± 41.0 months (mean ± SD; median 75.5; range 8–181); whereas among the 191 patients who died, the observed survival time was 37.8 ± 30.5 months (mean ± SD; median 26.0; range 6–130). The shorter observed times among deceased patients reflect death as the terminating event rather than incomplete surveillance, consistent with the reverse Kaplan–Meier estimate of potential follow-up duration reported above. The baseline characteristics are summarized in Table 1.

Of the 156 patients with early-stage (I–II) disease, 104 (66.7%) received adjuvant radiotherapy; this rate is explained principally by margin status rather than by stage-inappropriate treatment intensification. Among these 104 irradiated early-stage patients, 81 (77.9%) had a close (*n* = 58) or positive (*n* = 23) final margin, meeting the pre-specified indication for adjuvant radiotherapy described in Section 2.2, while the remaining 23 (22.1%) had a clear margin and were irradiated for another guideline-based risk factor (e.g., PNI or LVI).

### 3.2. Univariate Analysis

Per univariate Cox regression, CCI was significantly associated with OS (HR 1.14 per point, 95% CI 1.04–1.25, *p* = 0.005), as were age (HR 1.02, 95% CI 1.00–1.03, *p* = 0.007), advanced stage (HR 1.92, 95% CI 1.44–2.57, *p* < 0.001), PNI (HR 1.71, 95% CI 1.13–2.57, *p* = 0.010), LVI (HR 2.23, 95% CI 1.40–3.56, *p* < 0.001), and ENE (HR 2.02, 95% CI 1.43–2.86, *p* < 0.001). Sex was not associated with OS (HR 0.88, 95% CI 0.65–1.19, *p* = 0.409).

Notably, CCI showed no association with DFS (HR 1.02, 95% CI 0.92–1.12, *p* = 0.768), in contrast to advanced stage (HR 2.70, 95% CI 1.91–3.83, *p* < 0.001), PNI (HR 2.17, 95% CI 1.43–3.30, *p* < 0.001), LVI (HR 2.46, 95% CI 1.52–4.00, *p* < 0.001), and ENE (HR 1.99, 95% CI 1.37–2.91, *p* < 0.001), all of which remained strongly predictive of recurrence. This dissociation—CCI predicting OS but not DFS—is compatible with the hypothesis that the association between comorbidity and overall mortality may be driven predominantly by causes other than cancer recurrence. The results of the univariate analysis for both outcomes are shown in Table 2.

Because the primary analytic framework of this study is the early-versus-advanced-stage dichotomy underlying the CCI × stage interaction, Table 2 presents stage in this dichotomized form. For completeness, we additionally fit univariate Cox models for OS and DFS with stage retained as a four-level categorical variable (stage I as a reference; Supplementary Table S2). Relative to stage I, the hazard for OS increased progressively with stage (stage II: HR 1.07, 95% CI 0.68–1.68, *p* = 0.768; stage III: HR 1.65, 95% CI 1.07–2.53, *p* = 0.022; stage IV: HR 2.34, 95% CI 1.56–3.50, *p* < 0.001), and a similar pattern was observed for DFS (stage II: HR 0.87, 95% CI 0.49–1.55, *p* = 0.638; stage III: HR 1.95, 95% CI 1.17–3.24, *p* = 0.010; stage IV: HR 3.08, 95% CI 1.93–4.91, *p* < 0.001). This stage-by-stage gradient is consistent with the early/advanced dichotomy used in the primary analysis, and shows that most of the incremental risk is concentrated in stages III and IV relative to stage I, with stage II not significantly different from stage I for either endpoint.

### 3.3. Age–Comorbidity Collinearity

Age and CCI were highly correlated (Pearson r = 0.80, *p* < 0.001). A simple additive model containing both terms attenuated the CCI effect toward the null (HR 1.08, *p* = 0.26–0.35 across adjustment sets), which may reflect shared prognostic information between age and CCI and the reduced precision of the individual coefficients; however, residual confounding and model instability cannot be excluded (see Section 3.5).

### 3.4. Stage-Stratified Analysis

**Figure 1:**
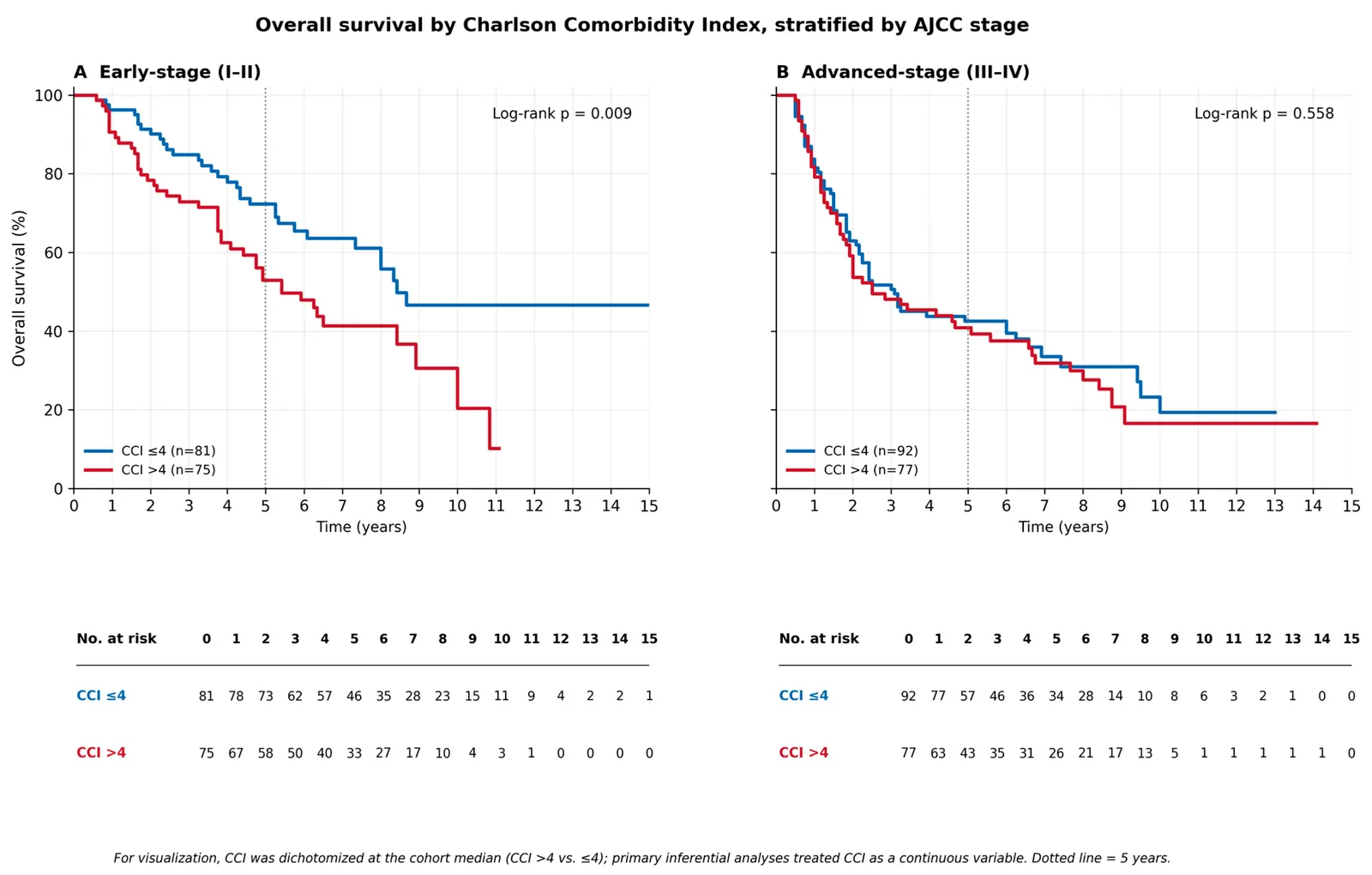
Overall survival by Charlson Comorbidity Index (CCI > 4 vs. ≤4), stratified by AJCC stage, with time axes in 1-year intervals and a number-at-risk table below each panel. (**A**) Early stage (I–II): log-rank *p* = 0.009. (**B**) Advanced stage (III–IV): log-rank *p* = 0.558. For visualization, CCI was dichotomized at the cohort median (CCI > 4 vs. ≤4). Primary inferential analyses treated CCI as a continuous variable. Dotted line = 5 years.

Within early-stage patients (*n* = 156), those with above-median CCIs (>4) had significantly worse 5-year OS than those with CCIs ≤ 4 (52.9% vs. 72.3%, log-rank *p* = 0.009). Within advanced-stage patients (*n* = 169), 5-year OS did not differ significantly with either CCI group (40.9% vs. 42.6%, log-rank *p* = 0.56). Kaplan–Meier curves for both subgroups are shown in Figure 1.

### 3.5. Formal Interaction Analysis

A multivariable Cox model for OS, including CCI, advanced stage, their interaction term, and age, confirmed a significant CCI × stage interaction (HR 0.82, 95% CI 0.68–0.99, *p* = 0.036), indicating that the hazard associated with each additional CCI point is significantly attenuated in advanced-stage disease relative to early-stage disease. In this model, the main CCI effect (representing its effect within early-stage patients) remained significant after age adjustment (HR 1.19, 95% CI 1.00–1.42, *p* = 0.047). The results were materially unchanged in a fully adjusted model that additionally included sex, PNI, LVI, and ENE (CCI main effect HR 1.20, *p* = 0.038; interaction HR 0.81, *p* = 0.034; Supplementary Table S1), while age itself was not significant in either interaction model (*p* = 0.275 and *p* = 0.133). The basic interaction model is shown in Table 3. Based on the model’s covariance matrix, the estimated CCI effect within advanced-stage disease was HR 0.98 per point (95% CI 0.83–1.15, *p* = 0.80), confirming no meaningful CCI–OS association among advanced-stage patients.

### 3.6. Recurrence Status Among Deaths

As a further direct test of the competing-mortality hypothesis, we classified each death according to whether the patient had a documented recurrence before death: deaths occurring in patients with a documented recurrence were classified as cancer-related, and deaths occurring in patients with no recorded recurrence at last follow-up were classified as attributable to other, non-cancer causes. Of 191 deaths, 130 (68.1%) were cancer-related and 61 (31.9%) were attributable to other causes. This split differed by stage: among early-stage patients who died (*n* = 75), 41 (54.7%) were cancer-related and 34 (45.3%) were attributable to other causes, whereas among advanced-stage patients who died (*n* = 116), 89 (76.7%) were cancer-related and only 27 (23.3%) were attributable to other causes. The substantially higher proportion of other-cause death among early-stage patients supports a relatively greater contribution of competing, non-oncologic mortality in this subgroup, consistent with the CCI × stage interaction reported above; the corresponding predominance of cancer-related death in advanced-stage patients is consistent with oncologic factors dominating the survival calculus once disease is advanced. Because the institutional database did not record an independently adjudicated cause of death (e.g., death certificate or autopsy confirmation), this classification is based on recurrence status rather than formal cause-of-death determination, and is noted as a limitation in Section 4.7.

Taken together, these results indicate that comorbidity burden, quantified by CCI, is significantly associated with worse overall survival specifically among patients with early-stage OCSCC, with no corresponding association evident in advanced-stage disease or with disease-free survival at either stage, supporting tumor stage as an effect modifier of the CCI–survival relationship. The recurrence-based classification of deaths (Section 3.6) provides direct quantitative support for this interpretation, showing that non-cancer deaths accounted for a substantially larger share of mortality in early-stage than in advanced-stage patients.

## 4. Discussion

In this retrospective cohort of 325 patients treated surgically for oral cavity squamous cell carcinoma (OCSCC), comorbidity burden, quantified using the Charlson Comorbidity Index (CCI), was significantly associated with overall survival (OS) but not with disease-free survival (DFS). Critically, this association was not uniform across disease extent; CCI predicted OS within early-stage disease but not within advanced-stage disease, and this stage-dependence was confirmed formally by a significant CCI × stage interaction term (HR 0.82, 95% CI 0.68–0.99, *p* = 0.036) that persisted after adjustment for age, sex, and adverse histopathologic features. Taken together, these findings suggest that the association between comorbidity and overall mortality may be driven predominantly by competing causes of death, and they identify the tumor stage as a potential effect modifier of the prognostic association between CCI and survival.

### 4.1. CCI Predicts Overall but Not Disease-Specific Survival: A Competing-Mortality Signature

The dissociation between CCI’s effect on OS and its null effect on DFS is the central empirical finding of this analysis, and it recapitulates a pattern reported broadly across oncology. In a meta-analysis of prostate cancer cohorts, Cui et al. found a pooled hazard ratio of 1.59 for all-cause mortality and 1.88 for other-cause mortality with elevated CCIs, but there was no meaningful association with cancer-specific mortality (pooled HR 0.98, 95% CI 0.90–1.08) [8]—essentially the same OS/DFS split observed here. Within head and neck cancer, a nationwide Danish case–control study of over 25,000 patients similarly evaluated comorbidity against overall and cancer-specific mortality as distinct endpoints [9], and a meta-analysis of laryngeal squamous cell carcinoma spanning 28 studies and over 32,000 patients found that CCI predicted overall but not disease-free survival [10]. This convergence is compatible with comorbidity burden influencing overall mortality predominantly through causes other than cancer recurrence; the recurrence-based classification of deaths presented in Section 3.6, in which non-cancer deaths accounted for 45.3% of early-stage deaths versus 23.3% of advanced-stage deaths, provides direct support for this mechanism within our cohort, although independently adjudicated cause-of-death data were not available to confirm it definitively.

### 4.2. Magnitude and Consistency with the Oral Cavity and Head and Neck Literature

The magnitude of the CCI–OS association here is consistent with prior oral cavity and head and neck cohorts. A recent comparison of comorbidity indices in a large head and neck oncology cohort reported 5-year survival of 62.0% for CCI of zero versus 21.2% for CCI of five or higher [16]—a gradient comparable to the 5-year OS difference we observed between low- and high-CCI groups within early-stage disease (72.3% vs. 52.9%). Ferreira et al. likewise identified comorbidity as a prognostic factor for survival in oral squamous cell carcinoma [15], and a population-based case–control study of OSCC patients matched to population controls found stepwise decreases in 5-year OS with an increasing CCI [14], directly paralleling our own dose-related findings in this tumor site. Outside the oral cavity, a Charlson–Deyo index analysis of sinonasal squamous cell carcinoma similarly demonstrated an independent impact of comorbidity on overall survival after surgery [17], reinforcing that this relationship is not idiosyncratic to a single anatomic subsite within the head and neck.

### 4.3. Tumor Stage as an Effect Modifier

The attenuation of CCI’s prognostic effect in advanced-stage disease is less consistently examined in the literature but is broadly compatible with existing work on comorbidity in advanced oral cancer. Using the POROMS database, Liu et al. analyzed 448 patients with stages III–IV OSCC and found that comorbidity’s impact operated largely through delaying diagnosis and constraining treatment rather than through a direct prognostic effect [20]—a mechanism distinct from, and complementary to, the direct survival effect we observed in early-stage disease—consistent with oncologic factors (stage, PNI, LVI, ENE) dominating the survival calculus once disease is advanced. A cross-tumor-type analogy is offered by a study of stages I/II non-small-cell lung cancer, which isolated comorbidity’s effect within an early-stage subgroup following thoracoscopic resection [21]; the analytic logic mirrors the approach taken here and lends indirect support to comorbidity’s prognostic visibility being greatest when oncologic risk is otherwise low.

### 4.4. Age–Comorbidity Collinearity

Age and CCI were strongly correlated in this cohort (r = 0.80), and a simple additive model containing both terms attenuated the CCI effect toward the null—a pattern that may reflect shared prognostic information and reduced precision of individual coefficients. Stage-stratified and interaction-based analyses were therefore used to characterize effect modification, though these do not eliminate the underlying age–CCI correlation. This relationship is well-recognized in the literature: a comparison of three comorbidity indices in 674 OSCC patients found that age-adjusted CCI outperformed the unadjusted index [18], and an analysis of laryngeal squamous cell carcinoma found that high age-adjusted CCI carried a markedly elevated mortality risk on multivariable analysis [19], a pattern also reported in esophageal squamous cell carcinoma after radical esophagectomy [27], suggesting much of CCI’s prognostic content is bound up with age. Interaction testing was used to evaluate stage-dependent heterogeneity, rather than to resolve age–CCI collinearity. Whether an age-adjusted formulation (ACCI) would outperform our stratified approach here is a reasonable question for future validation but was outside this study’s scope.

### 4.5. Clinical Implications for Treatment-Intensity Decision-Making

The stage-dependence of the association between CCI and survival may have clinical relevance. Comorbidity assessment may provide additional prognostic information in early-stage OCSCC, particularly for estimating life expectancy, perioperative risk, and expected absolute treatment benefit. In advanced-stage disease, established oncologic factors may dominate. The present retrospective data do not support treatment de-escalation or organ-preserving selection on the basis of CCI alone. This framing is consistent with precedent elsewhere in head and neck and oncologic surgery. CCI has been identified as one of the significant factors determining surgical candidacy among elderly patients with OSCC [22], and comorbidity indices have been shown to guide treatment-intensity decisions in other tumor types, including HER2-positive breast cancer in older women [23]. More recently, comorbidity profiling has been used to identify clinically distinct elderly subgroups within nasopharyngeal carcinoma, supporting the broader principle that comorbidity assessment can meaningfully stratify treatment-relevant risk groups rather than functioning only as a statistical covariate [24]. This is not a blanket argument for de-escalation in all comorbid patients; a recent analysis of elderly head and neck cancer patients treated with concomitant chemoradiotherapy found that careful patient selection preserved outcomes with standard-intensity therapy despite comorbidity [28], underscoring that comorbidity burden should inform rather than replace individualized treatment planning. Because comorbidity’s prognostic weight is concentrated in early-stage disease, where oncologic risk is otherwise low, we suggest CCI assessment may be most clinically useful in this subgroup for two practical purposes: guiding the intensity of postoperative surveillance and general-health follow-up (e.g., more frequent internal medicine or geriatric input for high-CCI patients whose overall survival is disproportionately threatened by comorbidity rather than recurrence, consistent with evidence that frailty and geriatric assessment independently inform survival and treatment tolerance in older head and neck cancer patients [29]); and informing preoperative medical optimization before curative-intent surgery, when comorbidities have a more direct bearing on long-term survival than in advanced-stage patients whose prognosis is dominated by oncologic factors.

### 4.6. Methodological Considerations

Two analytic choices departed from default CCI application. First, we excluded points allocated to the index malignancy, so the score reflects comorbid disease independent of the index cancer. This mirrors a concern raised by Borrelli et al. (2023), who found that only two out of 115 observational cancer studies had applied CCIs matching their original validation outcomes, and the cancer type matched the validation population in only 16 of 115 [6]—an argument against off-the-shelf application and for deliberate disease-specific scoring decisions. This concern is echoed by the index’s original developer, Charlson et al. (2022), in a critical review of its clinimetric properties [7]. Second, CCI was retained as continuous in all primary analyses; dichotomization at the cohort median (>4 vs. ≤4) was used only for graphical presentation of Kaplan–Meier curves, avoiding inferential weight on a data-derived threshold. We also note that newer diagnosis-based comorbidity indices have demonstrated improved prediction of death relative to CCI in other cancer populations [30]; whether such measures would outperform CCI in OCSCC remains untested here.

CCI was analyzed exclusively as a composite score, consistent with its standard clinical and research application. We did not examine whether individual comorbidity categories (e.g., cardiovascular disease, diabetes, chronic pulmonary disease) contributed disproportionately to the OS association, because our institutional database recorded comorbidity only as the aggregate CCI score and a binary indicator of any chronic disease, without retaining the individual diagnostic categories underlying each patient’s score. We therefore cannot determine from the present data whether the CCI–OS association is driven broadly across comorbidity categories or disproportionately by one or a few specific conditions; this is an important question for future work using databases that capture comorbidity at the individual-diagnosis level, and we have added this point to Section 4.7.

### 4.7. Limitations

This study has several limitations. It is a retrospective, single-institution analysis spanning a 12-year accrual period (2009–2021), during which staging conventions, surgical techniques, and adjuvant therapies may have evolved; thus, the era was not modeled directly. CCI was assessed only at diagnosis, without capture of comorbidity severity, functional status, or trajectory over treatment and follow-up. The strong age–CCI correlation, while addressed through stratified and interaction-based modeling, cannot be fully disentangled from age with the available data, and residual confounding cannot be excluded. Median follow-up, estimated using the reverse Kaplan–Meier method, was 89 months (95% CI 80–97); the shorter median observed times using a naive calculation (49 months) reflect death, which acts as a competing risk for extended observation rather than genuinely short surveillance. The positive- and close-margin rates observed here (22.8% and 51.7%, respectively) warrant explicit comment, as reported positive-margin rates in OCSCC vary widely with case mix. Large national registries dominated by earlier T-category disease report lower rates (e.g., 16.1% across 60,695 patients in the U.S. National Cancer Database, and 5.5% in a large Taiwanese cohort of 18,416 patients restricted to margins classified as <1 mm or frankly positive), whereas cohorts enriched for T3–T4 disease report rates similar to or higher than ours (18.1% in a National Cancer Database analysis restricted to cT3–T4 tumors; historical data showing positive-margin rates rising from 21% in T1 to 55% in T4 tumors). Most directly relevant, this rate closely replicates our own institution’s prior report on this identical surgical cohort (23.0% positive, 51.5% close margins) [25], indicating that the distribution is a reproducible feature of this population rather than a data or reporting artifact. Anatomically, 43.6% of tumors in this cohort arose in mandible-adjacent subsites (floor of mouth, 34.0%; mandibular gingiva, 6.4%; retromolar trigone, 2.5%), where the deep margin is bounded by periosteum or bone; positive-margin rates in these subsites (25.2%, 52.4%, and 62.5%) were markedly higher than in the tongue (12.3%), consistent with the surgical literature identifying mandibular proximity as a principal anatomical constraint on achieving the intended 1 cm margin, since surgeons must balance oncologic clearance against the morbidity of segmental mandibulectomy. The 1 cm target described in Section 2.2 therefore reflects surgical intent at the time of resection rather than a guaranteed pathological outcome, and the observed margin distribution should be interpreted in light of this subsite composition. Finally, this cohort is identical to that in our previously published analysis of surgical margins in this population [25], which examined a different exposure and did not address the CCI × stage question here; it also fully contains a non-tongue subset (*n* = 168) analyzed separately with respect to lymph node ratio in a manuscript currently under review (unpublished data); both overlaps are disclosed in the Data Availability Statement below and will be disclosed to the editor at submission. Margin status and treatment variables (postoperative radiotherapy and chemoradiotherapy) were not included in the primary model. A sensitivity analysis incorporating these confirmed the main CCI effect (HR 1.21, *p* = 0.036) and CCI × stage interaction (HR 0.74, *p* = 0.003); positive-margin status was independently associated with worse OS (HR 1.87, *p* = 0.004), while RT and chemoradiotherapy were not significant once stage and margin were accounted for. As a further sensitivity analysis, we fit a multivariable OS model beginning with a broader set of 14 candidate baseline clinical and pathologic covariates (age, sex, smoking status, alcohol use, PNI, LVI, ENE, tumor grade, margin status, adjuvant RT, adjuvant CRT, platelet-to-lymphocyte ratio, plus centered CCI, advanced stage, and their interaction) and applying backward elimination, retaining covariates significant at *p* < 0.05 (*n* = 322 with complete data for all candidate variables). The CCI × stage interaction remained significant in this more heavily adjusted, data-driven model (HR 0.78, 95% CI 0.64–0.94, *p* = 0.008), reinforcing the robustness of the primary finding; the retained model additionally included age, alcohol use, PNI, and margin status as independent predictors of OS, while the CCI main effect attenuated to non-significance (HR 1.13, 95% CI 0.94–1.36, *p* = 0.18) once this broader covariate set was included. Full The full results are provided in Supplementary Table S3.

Several further limitations warrant mentioning. First, this is a single-institution cohort of moderate size (*n* = 325) accrued over a long, 12-year enrollment window (2009–2021); the sample size is modest relative to large nationwide or multi-institutional registries, which affords these studies greater power to detect smaller effects and to model stage- or subsite-specific subgroups, and our estimates—particularly the interaction term—should be interpreted with this precision limitation in mind pending external validation in a larger, ideally multi-institutional or nationwide, cohort. Relatedly, because this analysis included only patients selected for, and able to undergo, primary surgery (*n* = 325), it is subject to selection bias relative to nationwide reference cohorts that also capture non-surgical patients; patients with advanced disease and substantial clinical frailty may not have been considered surgical candidates and are therefore not represented in this cohort. Our findings, including the attenuated CCI–OS association within advanced-stage disease, consequently apply to the population eligible for curative-intent surgical resection and may not generalize to all patients presenting with advanced oral cavity cancer, including those managed non-surgically because of comorbidity-related frailty. Second, we could not determine whether the attenuated CCI effect in advanced-stage disease partly reflects the dilution of adjuvant treatment benefit due to toxicity, dose reduction, or early discontinuation among frail, high-CCI patients, as our database did not capture treatment toxicity, dose-intensity, or reasons for treatment modification; this remains a plausible contributing mechanism alongside the competing-mortality interpretation proposed above. Third, causes of death were not independently adjudicated by death certificate or autopsy; deaths were classified as cancer-related versus other-cause based on documented recurrence status (Section 3.6)—a clinically standard approach in the absence of formal cause-of-death data—but one that could misclassify the rare patient who died of unrecognized or undocumented recurrence, or who had a recorded recurrence that was not the proximate cause of death. Finally, comorbidity was captured only as an aggregate CCI score, without retention of the individual comorbidity categories (e.g., cardiovascular disease, diabetes, chronic pulmonary disease) contributing to each patient’s score, so we could not assess whether the CCI–OS association is broadly distributed across comorbidity types or driven disproportionately by specific conditions. In addition, our DFS endpoint pooled true locoregional/distant recurrences (*n* = 132) with second primary malignancies (*n* = 15); although both events end the disease-free interval, second primary tumors are a biologically distinct outcome from recurrence of the index cancer, with their own, partly independent risk factors [31], and pooling them could in principle dilute a CCI–recurrence association if one were present.

## 5. Conclusions

Comorbidity burden, measured by CCI, was associated with overall but not disease-free survival after surgery for OCSCC, a pattern compatible with the hypothesis that comorbidity predominantly influences mortality through pathways other than cancer recurrence; a recurrence-based classification of deaths lent direct support to this interpretation, though independently adjudicated cause-of-death data were not available. This association was most apparent in early-stage disease and was substantially attenuated in advanced-stage disease, supporting stage-dependent heterogeneity in the prognostic relevance of CCI. Comorbidity assessment may therefore add prognostic information in early-stage OCSCC but should not be used in isolation to determine treatment intensity. External validation of the CCI × stage interaction, and prospective evaluation of comorbidity-informed treatment selection in early-stage OCSCC, represent logical next steps.

## Figures and Tables

**Table 1:** Baseline patient, tumor, and treatment characteristics (*n* = 325). All T and N categories reflect pathological staging (pT/pN) based on the surgical specimen, per AJCC criteria; AJCC overall stage (I–IV) is grouped from pT/pN/M in the standard manner. CCI, Charlson Comorbidity Index; SD, standard deviation; KM, Kaplan–Meier; CI, confidence interval; PNI, perineural invasion; LVI, lymphovascular invasion; ENE, extranodal extension; RT, radiotherapy; CRT, chemoradiotherapy. Early-(I–II) and advanced-stage (III–IV) groupings, used for the CCI × stage interaction analysis, are reported in the main text (Section 3.1) rather than as separate summary rows here, as they are directly derivable from the individual stage counts shown. Depth of invasion is not shown because it was not systematically available for patients treated before 2018 (see Section 2.2) and staging was therefore performed per AJCC 7th edition criteria throughout. “Recurrences (any)” (*n* = 147) comprises 132 true locoregional/distant recurrences and 15 second primary malignancies; both are counted as DFS events (Section 2.3). Final margin status was classified using the three-tier system applied throughout this cohort (consistent with [25]): positive margin, tumor at the inked margin (<1.0 mm); close margin, 1.0–4.9 mm; clear margin, ≥5.0 mm. Adjuvant radiotherapy alone and adjuvant chemoradiotherapy are reported as mutually exclusive categories; chemoradiotherapy patients also received radiotherapy but are counted only once, under CRT.

Characteristic	Value (*n* = 325)
Male sex, *n* (%)	210 (64.6)
Age, years, mean ± SD	59.1 ± 12.1
CCI, mean ± SD (range; median)	4.48 ± 1.64 (2–9; 4)
Pathological T stage (pT), *n* (%)	
pT1, *n* (%)	99 (30.5)
pT2, *n* (%)	158 (48.6)
pT3, *n* (%)	50 (15.4)
pT4, *n* (%)	18 (5.5)
Pathological N stage (pN), *n* (%)	
pN0, *n* (%)	189 (58.2)
pN1, *n* (%)	58 (17.8)
pN2b, *n* (%)	55 (16.9)
pN2c, *n* (%)	14 (4.3)
pN3, *n* (%)	9 (2.8)
AJCC stage, *n* (%)	
Stage I, *n* (%)	77 (23.7)
Stage II, *n* (%)	79 (24.3)
Stage III, *n* (%)	73 (22.5)
Stage IV, *n* (%)	96 (29.5)
Tumor grade, *n* (%)	
Grade 1 (well-differentiated), *n* (%)	67 (20.6)
Grade 2 (moderately differentiated), *n* (%)	207 (63.7)
Grade 3 (poorly differentiated), *n* (%)	51 (15.7)
Final margin status, *n* (%)	
Clear margin (≥5 mm), *n* (%)	83 (25.5)
Close margin, *n* (%)	168 (51.7)
Positive margin, *n* (%)	74 (22.8)
Adverse histopathologic features, *n* (%)	
Perineural invasion (PNI), *n* (%)	40 (12.3)
Lymphovascular invasion (LVI), *n* (%)	26 (8.0)
Extranodal extension (ENE), *n* (%)	56 (17.3) (*n* = 324; 1 missing value)
Adjuvant treatment, *n* (%)	
Adjuvant radiotherapy alone (RT), *n* (%)	178 (54.8)
Adjuvant chemoradiotherapy (CRT), *n* (%)	85 (26.2)
No adjuvant radiotherapy, *n* (%)	63 (19.3)
Follow-up and outcomes	
Median follow-up (reverse KM), months (95% CI)	89 (80–97)
Deaths, *n* (%)	191 (58.8)
Recurrences (any), *n* (%)	147 (45.2)
Local recurrence, *n* (%)	53 (16.3)
Regional (neck) recurrence, *n* (%)	33 (10.2)
Distant metastasis, *n* (%)	21 (6.5)
Locoregional recurrence, *n* (%)	17 (5.2)
Mixed recurrence, *n* (%)	8 (2.5)
Second primary, *n* (%)	15 (4.6)

**Table 2:** Univariate Cox proportional hazards regression for overall survival (OS) and disease-free survival (DFS), *n* = 325 (*n* = 324 for ENE-containing rows; one missing value excluded). CCI, Charlson Comorbidity Index; PNI, perineural invasion; LVI, lymphovascular invasion; ENE, extranodal extension; HR, hazard ratio; CI, confidence interval. Stage is shown dichotomized (advanced vs. early) to align with the primary early/advanced framework used throughout this study for the CCI × stage interaction; univariate HRs for the individual AJCC stages (II, III, and IV, each vs. stage I as a reference) are provided in Supplementary Table S2. Sex was not evaluated for DFS, as unlike stage, PNI, LVI, and ENE, sex has no established biological association with locoregional or distant tumor recurrence in oral cavity cancer; it was therefore included only in the OS model, where sex-related differences in comorbidity burden and competing mortality are more plausible.

Variable	OS: HR (95% CI)	DFS: HR (95% CI)	*p* (OS/DFS)
CCI (per point)	1.14 (1.04–1.25)	1.02 (0.92–1.12)	0.005/0.768
Age (per year)	1.02 (1.00–1.03)	1.01 (0.99–1.02)	0.007/0.479
Advanced stage (vs. early)	1.92 (1.44–2.57)	2.70 (1.91–3.83)	<0.001/<0.001
PNI (yes vs. no)	1.71 (1.13–2.57)	2.17 (1.43–3.30)	0.010/<0.001
LVI (yes vs. no)	2.23 (1.40–3.56)	2.46 (1.52–4.00)	<0.001/<0.001
ENE (yes vs. no)	2.02 (1.43–2.86)	1.99 (1.37–2.91)	<0.001/<0.001
Sex (female vs. male)	0.88 (0.65–1.19)	—	0.409/—

**Table 3:** Multivariable Cox model for overall survival: CCI × stage interaction (basic model; *n* = 325). CCI, Charlson Comorbidity Index; HR, hazard ratio; CI, confidence interval. A fully adjusted model, additionally including sex, PNI, LVI, and ENE, yielded materially unchanged estimates and is provided in Supplementary Table S1.

Variable	HR (95% CI)	*p*
CCI (main effect, within early stage)	1.19 (1.00–1.42)	0.047
Advanced stage	4.87 (1.94–12.25)	<0.001
CCI × advanced stage (interaction)	0.82 (0.68–0.99)	0.036
Age (per year)	1.01 (0.99–1.03)	0.275

## Data Availability

The datasets generated and/or analyzed during the current study are available from the corresponding author on reasonable request, subject to institutional approval and applicable data protection regulations. The cohort is identical to that reported in our previously published analysis of surgical margins in this population (Szewczyk et al., Cancers 2024, 16, 1488 [25]), which examined margin status as the primary exposure of interest and did not address the CCI × stage question examined here. This cohort also fully contains a non-tongue subset (*n* = 168) analyzed separately with respect to the lymph node ratio in a manuscript currently under review by our group (unpublished data).

## Supplementary Materials

The following supporting information can be downloaded at https://www.mdpi.com/article/10.3390/cancers18172825/s1, Table S1: Fully adjusted multivariable Cox model for overall survival, including CCI × advanced-stage interaction, sex, PNI, LVI, and ENE; Table S2: Univariate Cox proportional hazards regression for OS and DFS, with AJCC stage entered as a four-level categorical variable (using stage I as a reference); Table S3: Backward-elimination multivariable Cox model for overall survival, incorporating an expanded set of 14 candidate baseline clinical and pathologic covariates; Table S4: Completed REMARK reporting checklist.
