# Supplementary material for: Comorbidity Burden and Survival After Primary Surgery for Oral Cavity Squamous Cell Carcinoma: Tumor Stage as an Effect Modifier
Source: Cancers (Basel). 2026 Sep 1;18(17):2825. doi: 10.3390/cancers18172825 (PMC13564748; doi:10.3390/cancers18172825)
Supplement: Supplementary file 1 [file cancers-18-02825-s001.zip › Supplementary Table S1.pdf]

Supplementary Table S1. Fully adjusted multivariable Cox model for overall survival

Variable	HR (95% CI)	p
CCI (main effect, within early-stage)	1.20 (1.01–1.44)	0.038
Advanced stage	4.22 (1.61–11.09)	0.003
CCI × advanced stage (interaction)	0.81 (0.67–0.98)	0.034
Age (per year)	1.02 (1.00–1.04)	0.133
Sex (female vs. male)	0.81 (0.58–1.11)	0.188
PNI (yes vs. no)	1.55 (1.00–2.41)	0.052
LVI (yes vs. no)	1.34 (0.79–2.28)	0.280
ENE (yes vs. no)	1.40 (0.93–2.10)	0.103

Additionally adjusted for sex, PNI, LVI, and ECS beyond the basic model shown in Table 3 of the main text. n = 324 (one ECS value missing, excluded). The CCI × stage interaction remains significant after full covariate adjustment (p = 0.034), confirming that stage modifies the CCI–survival association independent of PNI, LVI, and ENE.
