# Supplementary material for: Comorbidity Burden and Survival After Primary Surgery for Oral Cavity Squamous Cell Carcinoma: Tumor Stage as an Effect Modifier
Source: Cancers (Basel). 2026 Sep 1;18(17):2825. doi: 10.3390/cancers18172825 (PMC13564748; doi:10.3390/cancers18172825)
Supplement: Supplementary file 1 [file cancers-18-02825-s001.zip › Supplementary_Table_S2.pdf]

Supplementary Materials

Manuscript: cancers-4497197 — "Comorbidity Burden and Survival After Primary Surgery for Oral Cavity Squamous Cell Carcinoma: Tumor Stage as an Effect Modifier"

Table S2. Univariate Cox proportional hazards regression for overall survival (OS) and disease-free survival (DFS) with AJCC stage entered as a four-level categorical variable (n = 325).

Stage (vs. Stage I)	OS: HR (95% CI)	OS: p	DFS: HR (95% CI)	DFS: p
Stage I (reference)	1.00 (—)	—	1.00 (—)	—
Stage II	1.07 (0.68–1.68)	0.768	0.87 (0.49–1.55)	0.638
Stage III	1.65 (1.07–2.53)	0.022	1.95 (1.17–3.24)	0.010
Stage IV	2.34 (1.56–3.50)	<0.001	3.08 (1.93–4.91)	<0.001

HR, hazard ratio; CI, confidence interval; OS, overall survival; DFS, disease-free survival. Univariate Cox models fit with AJCC stage as a four-level categorical predictor, Stage I as the reference category. n = 325 for OS; n = 325 for DFS. These stage-specific estimates complement the early- (I–II) versus advanced-stage (III–IV) dichotomization used as the primary analytic variable throughout the main text (Table 2, Table 3), which aligns with the pre-specified CCI × stage interaction framework.

Analysis note: models were fit using the survival package in R (or an equivalent Cox proportional-hazards implementation), consistent with the statistical methods described in Section 2.3 of the main text.
