# Supplementary material for: Comorbidity Burden and Survival After Primary Surgery for Oral Cavity Squamous Cell Carcinoma: Tumor Stage as an Effect Modifier
Source: Cancers (Basel). 2026 Sep 1;18(17):2825. doi: 10.3390/cancers18172825 (PMC13564748; doi:10.3390/cancers18172825)
Supplement: Supplementary file 1 [file cancers-18-02825-s001.zip › Supplementary_Tables_S3_S4.pdf]

Supplementary Materials

Manuscript: cancers-4497197 — "Comorbidity Burden and Survival After Primary Surgery for Oral Cavity Squamous Cell Carcinoma: Tumor Stage as an Effect Modifier"

Table S3. Backward-elimination multivariable Cox model for overall survival (n = 322).

Starting covariate set (14 candidates): centered CCI, advanced stage, CCI × advanced-stage interaction, age, sex, smoking status, alcohol use, perineural invasion (PNI), lymphovascular invasion (LVI), extranodal extension (ENE), tumor grade (poor vs. well/moderate), margin status (positive vs. free/close), adjuvant radiotherapy, adjuvant chemoradiotherapy, and platelet-to-lymphocyte ratio. Backward elimination retained covariates significant at $p < 0.05$; CCI, advanced stage, and their interaction were forced into the model regardless of significance, consistent with the primary analytic framework of this study.

Variable	HR (95% CI)	p
CCI (centered, per point)	1.13 (0.94–1.36)	0.179
Advanced stage (vs. early)	1.81 (1.33–2.46)	<0.001
CCI × advanced stage (interaction)	0.78 (0.64–0.94)	0.008
Age (per year)	1.03 (1.00–1.05)	0.022
Alcohol use (yes vs. no)	1.78 (1.18–2.69)	0.006
Perineural invasion (yes vs. no)	1.78 (1.17–2.72)	0.007
Positive margin (vs. free/close)	2.10 (1.53–2.89)	<0.001

HR, hazard ratio; CI, confidence interval; CCI, Charlson Comorbidity Index. Variables eliminated during backward selection (in removal order, with p-value at removal): adjuvant radiotherapy (p = 0.626), tumor grade (p = 0.310), lymphovascular invasion (p = 0.262), adjuvant chemoradiotherapy (p = 0.156), smoking status (p = 0.095), extranodal extension (p = 0.106), sex (p = 0.055), platelet-to-lymphocyte ratio (p = 0.126). Model concordance index = 0.673. This analysis complements, and is consistent with, the pre-specified primary and fully adjusted interaction models reported in Table 3 and Supplementary Table S1 of the main text: the CCI × stage interaction remains statistically significant across all three specifications.

Table S4. REMARK reporting checklist.

Completed per the REMARK (REporting recommendations for tumor MARKer prognostic studies) guideline. Section/item numbers follow the original REMARK publication (McShane et al., 2005) and its 2012 explanation and elaboration.

Item	REMARK Item Description	Reported in
1	State study objectives, prospectively defined hypotheses.	Abstract; Section 1.1 (Aim)
2	Describe characteristics of study patients, including source of patients.	Section 2.1
3	Describe treatments received and clinical/pathological characteristics.	Section 2.2 (full protocol documented: staging edition, margin criterion, frozen-section practice, neck dissection extent, MDT process, RT/CRT protocol and indications, database lock and analysis dates); Table 1
4	Specify type of study design; state whether prospective/retrospective; describe method of patient identification.	Section 2.1
5	State how tumor markers/predictors were measured, when, and by whom.	Section 2.3; Section 2.1 (2 investigators, B.W. and B.N.)
6	Specify definitions of outcomes (OS, DFS) and how and when assessed.	Section 2.3
7	List candidate variables considered for modeling.	Section 2.4; Supplementary Table S3
8	Describe statistical methods for model building, variable selection, and assumption testing.	Section 2.4
9	Report distribution of demographic/clinical characteristics; number of missing values.	Table 1; Section 3.1
10	Report follow-up: median/range, and method used (e.g., reverse Kaplan–Meier).	Section 3.1
11	Report survival estimates and effect sizes with confidence intervals, both univariate and multivariable.	Tables 2–3; Sections 3.2–3.5; Supplementary Tables S1–S3
12	Present results of all variable selection procedures, including those not statistically significant.	Section 3.5; Supplementary Table S3 (elimination order and p-values reported)
13	For each analysis, report number of events, sample size, and events/variable ratio if relevant.	Section 3.1; Table 1; figure legends
14	Discuss limitations, including potential bias and generalizability.	Section 4.7
15	Interpret results in the context of the totality of evidence; avoid overinterpretation.	Section 4 (Discussion), Sections 4.1–4.5
